# Development and Evaluation of an Antimicrobial Formulation Containing *Rosmarinus officinalis*

**DOI:** 10.3390/molecules27165049

**Published:** 2022-08-09

**Authors:** Lucas Malvezzi de Macedo, Érica Mendes dos Santos, Janaína Artem Ataide, Gabriela Trindade de Souza e Silva, João Paulo de Oliveira Guarnieri, Marcelo Lancellotti, Angela Faustino Jozala, Paulo Cesar Pires Rosa, Priscila Gava Mazzola

**Affiliations:** 1 School of Medical Sciences, University of Campinas (UNICAMP), Campinas 13083-888, Brazil; 2 Faculty of Pharmaceutical Sciences, University of Campinas (UNICAMP), Campinas 13083-871, Brazil; 3 Laboratory of Industrial Microbiology and Fermentation Process (LAMINFE), University of Sorocaba, Sorocaba 18023-000, Brazil

**Keywords:** rosemary, antioxidant, antimicrobial, cell viability, formulation

## Abstract

*Rosmarinus officinalis* belongs to the Lamiaceae family, and its constituents show antioxidant, anti-inflammatory, antidepressant, antinociceptive, and antibacterial properties. The aim of this study was to develop a topical formulation with *R. officinalis* extract that had antimicrobial and antioxidant activity. Maceration, infusion, Soxhlet, and ultrasound were used to produce rosemary extracts, which were submitted to antioxidant, compound quantification, cell viability, and antimicrobial assays. Infusion and Soxhlet showed better results in the DPPH assay. During compound quantification, infusion showed promising metabolite extraction in phenolic compounds and tannins, although maceration was able to extract more flavonoids. The infusion and ultrasound extracts affected more strains of skin bacteria in the disk diffusion assays. In the minimum inhibitory concentration assay, the infusion extract showed results against *S. aureus*, *S. oralis,* and *P. aeruginosa*, while ultrasound showed effects against those three bacteria and *E. coli*. The infusion extract was chosen to be incorporated into a green emulsion. The infusion extract promoted lower spreadability and appropriated the texture, and the blank formulation showed high levels of acceptance among the volunteers. According to the results, the rosemary extract showed promising antioxidant and antimicrobial activity, and the developed formulations containing this extract were stable for over 90 days and had acceptable characteristics, suggesting its potential use as a phytocosmetic. This paper reports the first attempt to produce an oil-in-water emulsion using only natural excipients and rosemary extract, which is a promising novelty, as similar products cannot be found on the market or in the scientific literature.

## 1. Introduction

Plant secondary metabolites with a pharmacological effect are used to develop topical formulations to deliver their effects into the skin for the treatment of local disorders [1]. For example, rosemary extract has shown antimicrobial activity in different cases [2,3,4,5,6]. Medicinal plants are widely used as food and improve human health due to their great number of bioactive compounds [7,8] Considering that the use of natural products is safe and economically viable, it is interesting to develop products for topical diseases using these extracts. 

Extracts of *Rosmarinus officinalis*, which belongs to the *Lamiaceae* family and is popularly known as rosemary [9], have been used for antioxidant, anti-inflammatory, antidepressant, antinociceptive, and antibacterial purposes [10,11,12,13]. The review article published by de Macedo, et al. [14] revisited a range of its medicinal (anti-inflammatory, skin cancer, wound healing, antimicrobial, skin flap survival, transdermal drug delivery, and antifungal) and cosmetic (ginoid lipodystrophy, alopecia, antiaging, and ultraviolet protection) properties in vitro and in vivo. The secondary metabolites of rosemary that are responsible for these therapeutic activities were identified as flavonoids, polyphenols, and terpenes [15,16,17,18] and were identified by chromatographic techniques [18,19].

The concentration of bioactive compounds changes according to the extraction method used. The rosmarinic acid concentration varies when subjected to different extraction methods (maceration with stirring, heat reflux, and microwave-assisted extraction) and conditions (solvent, temperature, and time) [20]. Biological activities also change depending on the type of extraction method used. Ultrasound, solid–liquid, and supercritical fluid extraction showed different results when applied to phenolic compounds, antioxidant activity, the minimum inhibitory concentration, and the minimum bactericidal concentration [21].

The aim of this study was to develop a topical formulation (oil-in-water) using *Rosmarinus officinalis* extract that had antimicrobial activity. To this end, four extraction methods were used, and the rosemary extracts were evaluated for in vitro compound quantification as well as for antioxidant and antimicrobial activity. The chosen extract was then incorporated into a topical formulation, which was characterized and analyzed for stability.

## 2. Results

### 2.1. Antioxidant Activity

Four different extraction methods were used to obtain rosemary extracts: maceration (MAC), infusion (INF), Soxhlet (SOX), and ultrasound (ULT). The antioxidant activities of the extracts were then evaluated by the DPPH and FRAP assays.

**Figure 1:**
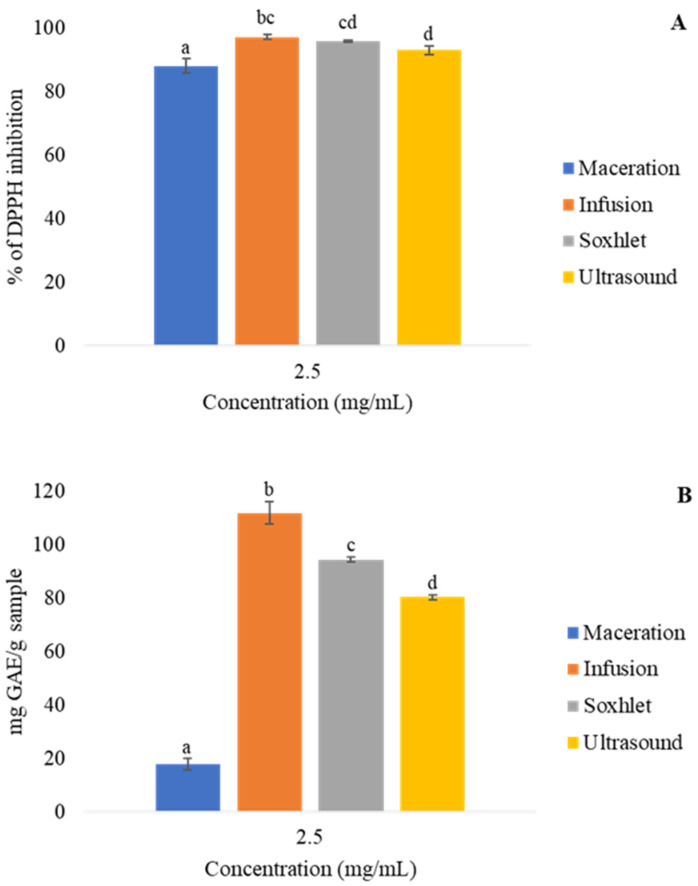
Antioxidant activities in DPPH (**A**) and FRAP (**B**) assays of rosemary extracts obtained from maceration, infusion, Soxhlet, and ultrasound. Results are presented as average ± standard deviation, *n* = 3. The letters represent significant differences between samples.

The effective concentration to inhibit 50% (EC_50_) of the radicals in the antioxidant assays is shown in Table 1, and the antioxidant activity expressed as percentages of inhibition and the gallic acid equivalent for the DPPH and FRAP assays, respectively, are shown in Figure 1.

According to Figure 1A, the *R. officinalis* extracts obtained by the INF and SOX techniques showed a higher potential to inhibit DPPH radicals. The INF and ULT samples showed better EC_50_ results than the others. 

According to Figure 1B, infusion showed the best FRAP results (111.94 ± 4.26 mg GAE/g), with a significant difference being observed compared to the other samples. 

### 2.2. Phenol, Flavonoid and Tannin Determination

**Figure 2:**
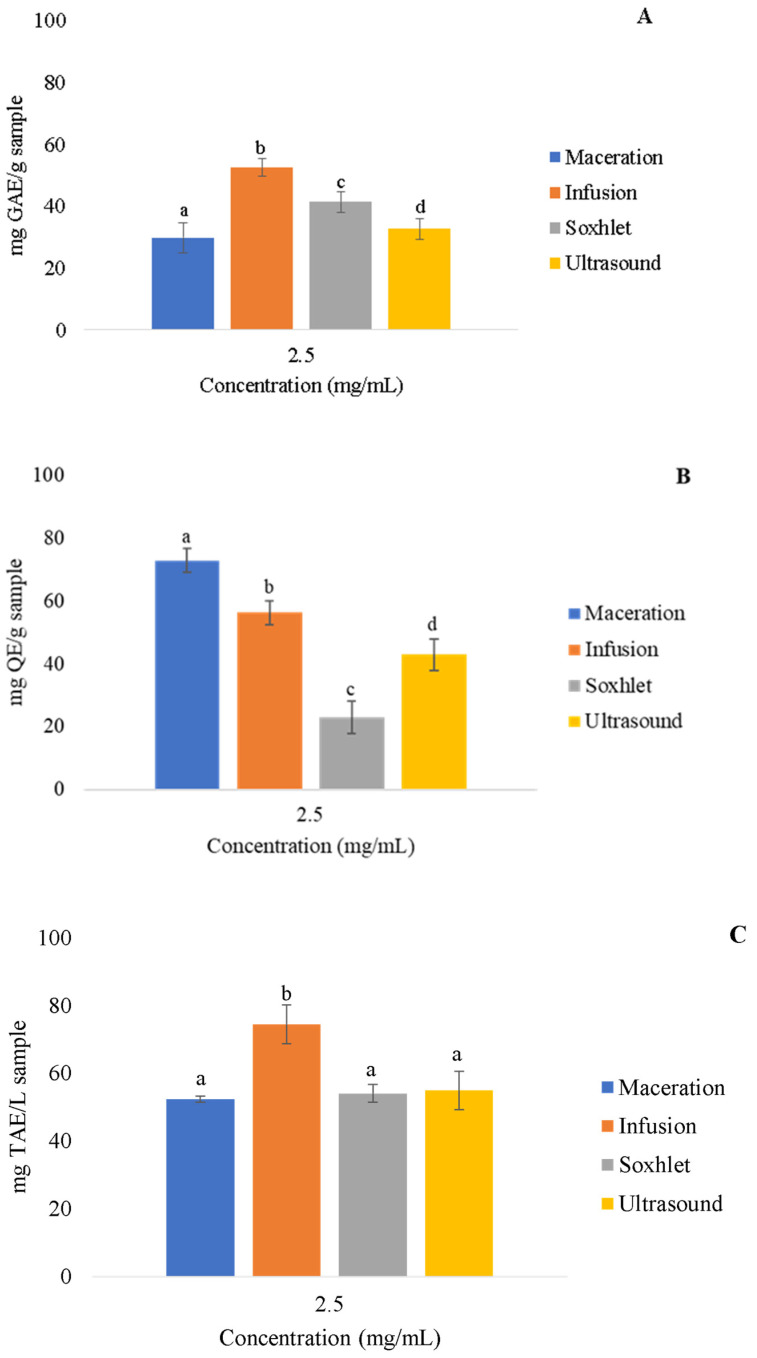
Determination of total phenolic compounds (**A**), flavonoids (**B**), and tannins (**C**) of samples of rosemary extracts obtained by maceration, infusion, Soxhlet, and ultrasound at a 2.5 mg/mL concentration in mg equivalent/g. Results are presented as average ± standard deviation, *n* = 3. The letters represent significant differences between samples.

After the antioxidant activity measurements, the four *Rosmarinus officinalis* extracts (2.5 mg/mL) were also characterized by determining the concentration of secondary metabolites according to the concentrations of phenolic compounds, flavonoids, and tannins. The obtained results are shown in Figure 2 and are expressed as standard equivalents. In the phenolic compound assay, the extract obtained by INF showed 52.50 ± 2.75 mg GAE/g, which was the largest phenol concentration compared to the other samples. 

The maceration extract showed 72.88 ± 3.84 mg QE/g, and maceration was considered the best extraction method for flavonoids. All of the samples showed significant differences. INF showed better results for tannin extraction, with 74.47 ± 8.73 mg TAE/g, which was significantly higher than all of the other methods.

### 2.3. Cell Viability Assay

**Figure 3:**
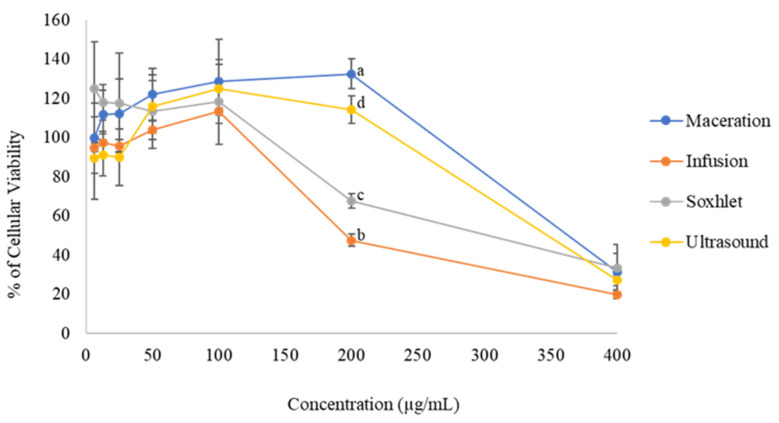
Cell viability percentage of rosemary extract in HaCaT cells obtained by maceration, infusion, Soxhlet, and ultrasound at 400, 200, 100, 50, 25, 12.5, and 6.25 µg/mL concentrations. Results are presented as average ± standard deviation, *n* = 3. The letters represent significant differences between samples.

Besides the composition and antioxidant activity of the extracts, their safety was evaluated in vitro in HaCaT cells. The cell viability percentage is shown in Figure 3. The IC50 values for INF, SOX, and ULT were 193.3, 193.4 and 235.2 µg/mL, respectively. The MAC value was inconclusive.

The concentration of 400 µg/mL showed less cell viability for all of the samples. Based on the results, it can be considered that the safest extracts were those with concentrations ranging from 6.25 to 100 µg/mL. In addition, some values exceeded the rate of 100%, which could be an indication that the extracts stimulate cell proliferation. According to ISO10993 [22], samples that reach at least 70% viability are considered nontoxic.

### 2.4. Microbiological Activity

#### 2.4.1. Disk Diffusion Screening

Antimicrobial activity has also been reported in rosemary extracts in the scientific literature, and thus, a screening to determine antimicrobial activity was performed using the disk diffusion method and microorganisms isolated from hospital samples. The 100% ethanol extract preparation used did not show interference with the antimicrobial activity. On the other hand, pen-strep, which was used as a positive control, proved to be more effective against a large number of bacteria than the tested rosemary extracts.

According to the findings, the samples showed different results. The results were dependent on the type of microorganism and the origin of the sample. For *P. aeruginosa* from a catheter sample, all of the extracts showed antimicrobial activity (halos above 10 mm). However, for *P. aeruginosa* from a urine sample, only SOX was unable to inhibit the microorganism. The same behavior was observed for *Acinetobacter baumannii* and *A. hemolyticus* from catheter samples.

Even though all of the samples showed antimicrobial activity, the INF and ULT samples inhibited a greater number of microorganisms than SOX and MAC. The INF sample showed antimicrobial activity (halo above 10 mm) against *Enterobacter agglomerans* from urine and *Escherichia coli* from tracheal secretions. The ULT sample showed antimicrobial activity (halo above 10 mm) against *E. coli* from urine.

#### 2.4.2. Evaluation of Minimum Inhibitory Concentration (MIC)

**Figure 4:**
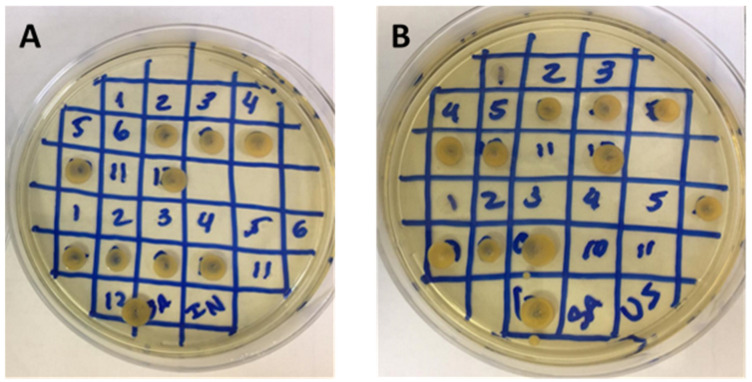
MIC assay of infusion (**A**) and ultrasound (**B**) extracts with different sample concentrations in a Petri plate. Microorganism growth was observed in diverse concentrations, as shown by the numbers. Number 12 is a positive control of microorganism growth.

Since INF and ULT inhibit the growth of a greater number of microorganisms, the MIC was evaluated using these extracts and standard microorganisms. The MIC results are displayed in Table 2 and Figure 4.

Both the INF and ULT extracts showed antimicrobial activity against the used microorganisms; however, differences were observed between them. The ULT extract produced an effect against all of the strains, but INF was two times more effective. These results could be related to the extraction technique, with a higher concentration of phenolic compounds, flavonoids, and tannins accounting for greater antioxidant activity and consequent antibacterial activity.

### 2.5. Formulation Development and Evaluation

#### 2.5.1. Phytocosmetic Development and Stability

Based on the previous results reported here, the infusion extract (INF) was chosen to be incorporated into an oil-in-water emulsion. Blank (BF) and *Rosmarinus officinalis* extract (EF) formulations were prepared and showed the physical aspects of a cream: shiny, creamy, and white in color with a characteristic odor from the base, although the rosemary extract induced a color change from white to yellowish. BF and EF showed no phase separation in a previous centrifuge test, pH values of 5.6 and 5.62, densities of 0.82 g/L and 0.84 g/L, and viscosities of 96,666.7 ± 11.015 cP and 106,666.7 ± 8,326.6 cP, respectively.

BF showed no phase separation during 90 days of assay. In all of the tested conditions, the formulation’s pH did not exceed 10% variation [23], but significant differences were reported when the formulation was compared to the initial sample at temperatures of 45 ± 2 °C and 5 ± 2 °C. The density and viscosity increased significantly and exceeded the limit of 10% variation [23] during the accelerated stability assessment under all conditions.

EF also showed no phase separation. The formulation presented no pH variation higher than 10% [23], and no significant differences were observed. Density and viscosity were compared to the first-day sample. The former parameter showed a significant increase over 90 days, and the latter parameter showed a significant increase at room temperature, although when submitted to a higher temperature, the formulation only showed a significant increase at day 7, and when stored in a refrigerator, a considerable increase in this parameter was observed after 30 days.

During the stability assessment, a darkening of the formulations’ color was observed when stored in a climatic chamber. Thus, it can be concluded that BF and EF showed an increase in the density and viscosity parameters during the accelerated stability assessment.

#### 2.5.2. Formulation Texture Analysis

Spreadability is the ability of semisolids to spread over a surface and is directly linked to viscosity and the type of polymer used in the formulation composition [24,25]. According to the results that were obtained (Table 3), the emulsions showed significant differences, with higher values of firmness and work of shear being obtained for EF. Considering the definition mentioned above, EF showed higher values for the parameters that were measured, spreading with more resistance than BF.

The texture analysis results are shown in Table 4. The cohesion test showed no significant differences. According to this finding, the *Rosmarinus officinalis* extract was responsible for increasing the formulations’ firmness, consistency, and cohesiveness.

#### 2.5.3. Formulation Sensorial Analysis

**Figure 5:**
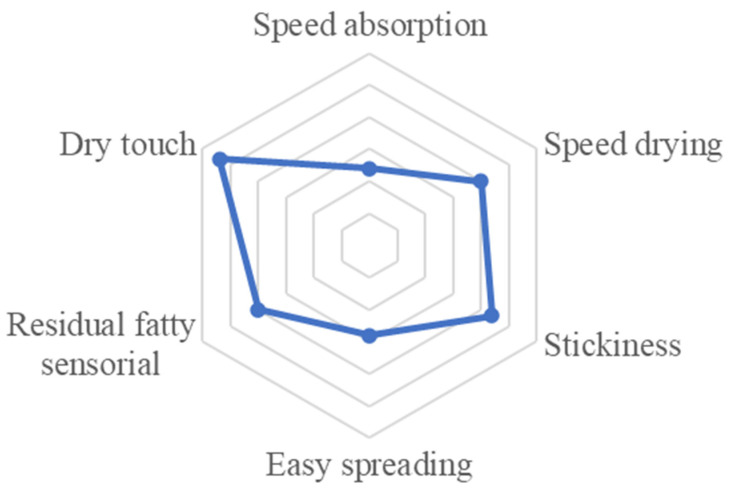
Sensorial evaluation of absorption speed, drying speed, stickiness, ease of spreading, residual fatty sensorial properties, and dry touch of the O/W emulsion.

Natural excipients were used in this formulation to create a new type of product. The sensorial analysis was carried out to understand the sensation of the cosmetic product on the volunteers’ skin and its level of acceptance. The results obtained are shown in Figure 5. After conducting the sensorial analysis with 50 volunteers (19 men and 31 women), it was observed that the mean values of each of the characteristics that were evaluated indicated that the product demonstrated high-speed drying, was dry to the touch, and had low stickiness and residual fatty sensorial properties, making it appropriate for use as a topical formulation. Conversely, it had a poor absorption speed and low spreadability. 

The formulation had an average overall ratio of 4.08 out of 5 points, and 42 participants expressed that they would use the product, indicating good acceptance.

## 3. Discussion

Secondary metabolites are chemical agents that are produced by plants as a means of survival [26]. These molecules have the ability to exert antioxidant, antimicrobial, and anti-inflammatory activities [27,28,29]. 

The antioxidant assays indicated that infusion and Soxhlet had higher DPPH inhibition potential and that infusion had higher iron-reducing action. The EC_50_ results for both techniques were lower than previous DPPH [30,31] and FRAP [32,33] findings. These lower values may be due to the type of solvent used, which may have had a higher affinity for antioxidant metabolites; the sample concentration for extract preparation; or the extraction method. 

The maceration and infusion techniques presented higher extractive efficiency of the phenolic compounds, with values of 51.11 ± 4.86 and 52.50 ± 2.75 mg EAG/g, respectively. Bianchin, et al. [34] used a 10 g sample of freeze-dried rosemary extract in 100 mL of ethanol in a water bath at 70 °C, placed it under stirring for 30 min, and obtained values of 46.48 ± 0.08 mg EAG/g, which are lower than those obtained by the two aforementioned methods. The same author also reported a total flavonoid content of 11.89 ± 0.58 mg EQ/g, which is lower than what was found for the four techniques used in this study, with maceration obtaining the highest amount of 72.88 ± 3.84 mg EQ/g. 

The total tannins were evaluated, and the infusion technique showed the highest amount extracted. In contrast, there are no studies in the literature that quantify the tannins in *Rosmarinus officinalis* extract. 

The extracts made using MAC, INF, SOX, and ULT and that had concentrations of 6.25–100 µg/mL showed higher cell viability than the minimum concentration required to be considered nontoxic according to ISSO 10993 [22]. In contrast, viability assays in HaCaT cells have not been reported for rosemary extract in the literature; thus, a comparison was made across the Lamiaceae family. A higher IC_50_ was reported for *Melissa officinalis* leaves by Moacă, et al. [35], which were considered to be safer when compared to the rosemary extracts obtained by the four different techniques previously mentioned in this study [35].

The antimicrobial activity of *R. officinalis* extracts was investigated alone and in association with mint extract and tocopherol [36]. The results indicated that rosemary was responsible for inhibiting bacterial growth and for decreasing the number of bacteria. The same antimicrobial effect was seen for the INF and ULT extracts, which showed an effect against bacteria in the disk diffusion test and against ATCC standard bacteria in the minimum inhibitory concentration test. 

The different techniques, maceration, infusion, Soxhlet, and ultrasound, showed different profiles for antioxidant and microbiological activity, the quantification of bioactive compounds, and cell viability. This finding is in line with previous results showing how different extraction methods impact biological activities and the concentration of secondary metabolites [20,21,37]. 

Based on the results presented above, an oil-in-water formulation incorporating the infusion extract was developed for topical application. The physical characteristics that the emulsions presented were creaminess, a glossy appearance, and a characteristic odor of the base; however, the rosemary extract was responsible for causing the color to darken from white to yellow.

During the stability analyses, BF and EF showed no phase separation, pH values of 5.6 and 5.62, densities of 0.82 g/L and 0.84 g/L, and viscosities of 96.666.7 ± 11.015 cP and 106.666.7 ± 8.326.6 cP, respectively. The pH values of both formulations remained within the range proposed by the Stability Guide for Cosmetic Products [23], but significant differences were reported for BF when comparing the sample on day 0 with the other days when it was stored in the climatic chamber and refrigerator. The density and viscosity showed a significant increase during the tests in both the white emulsion and in the one containing the extract. 

The sensory acceptability of the product without the extract was evaluated. BF was accepted by 84% of the volunteers and received a score of 4.08 in an overall analysis. This test is important for the development of new pharmaceutical and cosmetic forms, since the acceptance of the population is an important factor in the adhesion of the product fir both medicinal purposes and cosmetic purposes [37].

Considering the tests performed here, it can be said that the formulation with the extract of *Rosmarinus officinalis* has potential antimicrobial action and that it could be used as a phytocosmetic, but further studies are still needed. 

## 4. Materials and Methods

2,2-Diphenyl-1-picrylhydrazyl (DPPH) and quercetin (95% purity) were purchased from Sigma–Aldrich (São Paulo, Brazil), gallic acid and Folin–Ciocalteu reagent were purchased from Dinâmica Química Contemporânea Ltda. (São Paulo, Brazil), tannic acid and sodium carbonate were purchased from Êxodo Científica (Sumaré, São Paulo, Brazil), and 3-(4,5-dimethylthiazol-2-yl)-2,5-diphenyltetrazoluim (MTT), 2-(4-iodophenyl)-3-(4-nitrophenyl)-5-phenyltetrazolium chloride (INT), pen–strep, and 2,4,6-tris(2-pyridyl)-s-triazine (TPTZ) were purchased. All other reagents were analytical grade. Dried rosemary leaves were purchased from a popular market in Mogi Mirim (São Paulo, Brazil) and registered in the National System of Genetic Resource Management and Associated Traditional Knowledge (SisGen) under registration number AE2335F.

### 4.1. Extraction

Four extraction methods were applied in this study, although for purposes of comparability, 5 g of rosemary leaves and 100 mL of ethanol (EtOH) 100% were used for all of the extractions, and the extraction time was kept constant (30 min). The extraction methods employed were maceration (MAC, [38]), infusion (INF, [39]), Soxhlet (SOX, [40]), and ultrasound (ULT, [41]). After extraction and cooling (when necessary), the samples were filtered with a paper filter and stored in a refrigerator. Prior to lyophilization, EtOH was removed in an evaporator (Fisotam 802) for 1 h at 80 °C and at 150 rpm. Then, samples were placed in a lyophilizer (Lyostar 2, Linkam Instruments, Surrey, UK) and kept at −40 °C under 100 mTorr of vacuum for 4 h to freeze, and the temperature was then increased from −40 °C to 20 °C over the course of 137 h [42]. The lyophilized samples were kept in a refrigerator.

### 4.2. Antioxidant Activity

In vitro antioxidant activity was assayed by DPPH [43] and FRAP [44] with modifications.

For DPPH, a lyophilized extract solution was prepared using methanol (10 mg/mL), and further dilutions were mixed (2.5–10 mg/mL). In a 96-well microplate, DPPH solution (280 µL) and samples (20 µL) were added in triplicate. After 30 min of incubation, the sample absorbance was read using a spectrophotometer (Thermo Scientific, Multiskan Sky, Massachusetts, US) at a wavelength of 517 nm. In addition, the blank (methanol), sample blank (extract without reagent), and positive control (quercetin) were prepared. The radical DPPH inhibition percentage of the extracts was calculated by

$$\%=\frac{\mathrm{Control}\;\mathrm{DPPH}\;\mathrm{Abs}-\mathrm{Sample}\;\mathrm{Abs}}{\mathrm{Control}\;\mathrm{DPPH}\;\mathrm{Abs}}$$

For FRAP, 10 mg/mL samples were diluted to a concentration of 2.5 mg/mL in methanol. In a 96-well microplate, 265 µL of FRAP reagent solution, 20 µL of the samples, and 15 µL of ultrapure water were added, and the microplate was then incubated in the dark for 30 min, after which a spectrophotometer measurement was made at the 595 nm wavelength. The values were expressed in milligrams of gallic acid equivalent per gram of sample (mg GAE/g) according to the calibration curve [44].

For both tests, a blank (methanol), sample blank (extract without reagent), and positive control (quercetin) were measured.

### 4.3. Phenol, Flavonoid, and Tannin Determination

In vitro compound quantification was assayed using methods for phenolic compounds, flavonoids, and tannins. All of the samples were resuspended in methanol at concentrations ranging from 10 mg/mL to 2.5 mg/mL.

For the phenolic compounds, the methodology used by Santos, et al. [45] was used with modifications. In a 96-well microplate, 20 µL of sample was mixed with 180 µL of ultrapure water, 20 µL of 1 N Folin–Ciocalteu reagent, 20 µL of methanol, and 60 µL of 10% NaCO_2_. Measurements were made with a spectrophotometer at 760 nm after 20 min of incubation. The values were expressed in mg of gallic acid equivalent per sample gram (GAE/g).

For the flavonoids, the protocol used by Alves and Kubota [46] was followed. The spectrophotometer measurements were made at a wavelength of 425 nm. The results were expressed in milligrams of quercetin equivalent per sample gram (mg QE/L).

For the tannins, the protocol used by Shad, et al. [47] was followed. The spectrophotometer measurements were made at a wavelength of 725 nm. The results were expressed in milligrams of tannic acid equivalent per liter (mg TAE/L).

For the phenolic compounds, the flavonoid and tannin assays used rutin as a positive control. 

### 4.4. Cell Viability Assay

The cytotoxicity of the rosemary extracts was evaluated according to the methodology used by Machado, et al. [48]. Immortalized human keratinocytes (HaCaT) were cultivated in RPMI medium with 10% bovine fetal serum and were incubated at 37 °C and 5% CO_2_. After confluence, cells were trypsinized with 2.5 g/L trypsin/EDTA 0.2 g/L solution and added to a 96-well microplate. Each well received 1 mL of cell culture, and its final concentration was 1 × 10^6^ cells/mL. *Rosmarinus officinalis* samples were added into microplate wells at different concentrations (6.25–400 µg/mL) for 24 h of incubation. After medium removal, 100 µL of MTT (3-(4,5 dimethylthiazol-2-yl)-2,5-diphenyltetrazolium bromide) solution was added to the wells, and they were then incubated for 4 h at 37 °C and 5% CO_2_. Finally, the MTT reagent was removed, 100 µL of 100% ethanol was added, and spectrophotometer (Thermo Scientific, Multiskan Sky, Massachusetts, United States) measurements were made at 570 nm. The cell viability values were determined following the method proposed by Mosmann [49].

### 4.5. Microbiological Activity

#### 4.5.1. Disk Diffusion Screening

The disk diffusion test was performed according to Pereira, et al. [50], Andrews [51], Mostafa, et al. [52], with some modifications. Rosemary extracts filtered with a sterilized Millipore filter (0.22 µm) at a volume of 50 mg/mL and were added to a sterilized filter paper disk (8 mm in diameter). The medium was made by starting with the dispersion of 10.5 mL Mueller-Hinton agar in a sterilized Petri plate and a mixture that had been previously inoculated with 15 mL of a bacterial suspension (100 mL of medium/1 mL of 10^7^ CFU) to assure a medium concentration of 10^5^ CFU/mL. The disks with extracts and a disk with 50 µL of 100,000 UI positive control pen–strep (Sigma) solution were added to the agar. The plates were stored at 5 °C for 2 h and were then incubated for 24 h at 35 °C. The presence of halos allowed initial screening. Ethanol 100% was tested to evaluate interference in the test.

The bacteria from different biological samples used in the test were collected from Sorocaba Hospital (Table 5).

#### 4.5.2. Evaluation of Minimum Inhibitory Concentration (MIC)

To evaluate the inhibitory potential of the rosemary extracts, the classical successive dilution method (MIC) was adapted to 96-well plates. The strains used were determined by the Clinical and Laboratory Standards Institute (CLSI) with the modifications described below: Gram-positive (*Staphylococcus aureus* ATCC 10390 and *Streptococcus oralis* ATCC 9811) and Gram-negative (*Pseudomonas aeruginosa* ATCC 9721 and *Escherichia coli* ATCC 25922).

The MIC assay followed the method described by Mostafa, Al-Askar, Almaary, Dawoud, Sholkamy and Bakri [52], with some modifications. The TSB culture medium was applied at 100 µL, and rosemary extracts with a concentration of 50 mg/mL were applied as an initial sample. Then, 100 µL of the initial solution was added to a 96-well microplate, and serial dilutions (until 0.02 mg/mL) were subsequently made with TSB, starting in the first well. Furthermore, 10 µL of inoculum (0,5 McFarland ± 10^6^ CFU/mL) was added to the wells containing the extract dilutions, and the microplate was incubated for 24 h at 37 °C.

### 4.6. Formulation Development and Evaluation

#### 4.6.1. Phytocosmetic Development and Stability

All of the raw material was certified by the Associação de Certificação Instituto Biodinâmico (IBD) to obtain a formulation with only natural products.

Oil-in-water (O/W) formulations (Table 6) were developed following the protocol proposed by Cefali, et al. [53]. Xanthan gum was solubilized beforehand, and then benzoic acid, soy lecithin, and sorbitol were added to another beaker with hot water. The oil phase contained stearyl alcohol, sunflower oil, and sorbic acid and was stirred and heated to 70 °C. The aqueous phase was added to the oil phase, and after mixing, xanthan gum was added gradually. After emulsion cooling, the volume was completed with distilled water, and the pH was adjusted to 5.5–6.5 with sodium hydroxide. Two types of formulations were made: a blank solution (BF) and a solution with 5% (*w*/*w*) *Rosmarinus officinalis* extract (EF).

The formulations with and without extract were assessed following the Cosmetic Products Stability Guide [23]. The formulations were submitted to preliminary stability analysis to evaluate the physical properties (color and odor), pH, and phase separation for 15 consecutive days. Then, accelerated stability testing was performed at 1, 7, 15, 30, 60, and 90 days, evaluating the aspects previously mentioned; density was measured using a pycnometer, and viscosity was measured using a rotational viscosimeter (Brookfield, Mod LV-T, São Paulo, Brazil) at 1.5 rpm for 30 s using spindle 4 at 27 ± 2 °C [23,53]. Before the stability tests, 5 g of both BF and EF was submitted to phase separation evaluation in a centrifuge at 3000 rpm for 30 min. During the stability study, the formulations were stored at room temperature (with and without light exposure) and under hot (climatic chamber, 45 ± 2 °C) and cold (refrigerator, 5 ± 2 °C) conditions. In these evaluations, BF and EF were considered stable if the formulations did not present variation higher than 10% [23].

#### 4.6.2. Formulation Texture Analysis

The rheological properties of BF and EF were assessed with a texture analyzer (Stable Micro Systems TAXT plus, Surrey, UK). All of the parameters used in this analysis are shown in Table 7. The firmness and shear force of both formulations were evaluated in a spreadability test, where the emulsion was placed in a female cone and pressed down to eliminate air pockets. Firmness, consistency, cohesiveness, and work of cohesion were calculated and analyzed in a standard-sized back extrusion container (50 mm diameter) that was approximately 75% full.

#### 4.6.3. Formulation Sensorial Analysis

BF was submitted to sensorial analysis, which was approved by the Ethics Committee (number: 23197519.1.0000.5404) of the State University of Campinas. For this analysis, 0.1 g of emulsion was administered to the forearms of 50 volunteers. An evaluation was conducted using a questionnaire, with the aim of determining the best sensorial parameters (speed of absorption, speed of drying, stickiness, ease of spreading, residual fatty sensorial properties, dry touch, and general evaluation) according to a scale from 1 to 5 (bad, weak, reasonable, good, and very good, respectively). In addition, whether the volunteers were interested in using the developed product was evaluated [53].

### 4.7. Statistical Analysis

The assays were performed in triplicate, and the values were interpreted using ANOVA (*p* < 0.05) followed by Tukey’s test. The results were added to GraphPad Prism 5.0 (Dotmatics,, San Diego, US) for analysis, to make graphs, and to determine the effective concentration 50% (EC_5_0) and inhibitory concentration 50% (IC_50_) values.

## 5. Conclusions

This paper reports the first attempt to produce an oil-in-water emulsion using only natural excipients and rosemary extract, which is a promising novelty, as similar products cannot be found on the market or in the scientific literature. This study showed that the use of different techniques to obtain the extracts from rosemary resulted in higher antioxidant and antimicrobial activity due to the higher amounts of extracted phenolic compounds, flavonoids, and tannins, and the extracts showed no toxicity at low concentrations. However, the incorporation of the rosemary extracts showed an increase in the density and viscosity of the formulation during the 90 days of testing. Additionally, in the sensorial test, the blank formulation had good acceptance. Therefore, the topical formulation created using *Rosmarinus officinalis* is a promising candidate for use against microorganisms due to its antimicrobial activity. However, further studies should be conducted, including in vitro and in vivo evaluations of the developed formulation, to prove its application characteristics and claimed activities.

## Figures and Tables

**Table 1:** Effective concentration (mg/mL) to inhibit 50% of DPPH and FRAP radicals for rosemary extracts obtained by maceration (MAC), infusion (INF), Soxhlet (SOX), and ultrasound (ULT).

Sample	DPPH	FRAP
MAC	6.84	7.65
INF	7.73	4.97
SOX	9.38	5.00
ULT	6.83	5.01

**Table 2:** Minimum inhibitory concentration values obtained by microdilution in a 96-well microplate for infusion (INF) and ultrasound (ULT) rosemary extracts with an initial concentration of 50 mg/mL to 0.02 mg/mL.

MIC (mg/mL)				
Sample	*S. aureus*	*S. oralis*	*E. coli*	*P. aureginosa*
INF	≥1.5	≥6.0	-	≥6.0
ULT	≥3.0	≥12.5	≥25.0	≥12.5

**Table 3:** Textural evaluation of firmness (g) and work of shear (g.sec) of both blank (BF) and extract (EF) formulations.

Formulation	Firmness (g)	Work of Shear (g·s)
BF	149.70 ± 7.51 ^a^	134.61 ± 17.59 ^b^
EF	185.30 ± 2.30 ^b^	176.87 ± 8.08 ^b^

Results are presented as average ± standard deviation, *n* = 3. The letters represent significant differences between BF and EF samples.

**Table 4:** Textural evaluation of firmness (g), consistency (g·s), cohesiveness (g), and work of cohesion (g·s) for both blank (BF) and extract (EF) formulations.

Formulation	Firmness (g)	Consistency (g·s)	Cohesiveness (g)	Work of Cohesion (g·s)
BF	172.88 ± 2.56 ^a^	1817.18 ± 33.37 ^a^	−108.36 ± 2.25 ^a^	−886.22 ± 52.97 ^a^
EF	183.03 ± 5.83 ^b^	1912.78 ± 18.49 ^b^	−114.92 ± 2.65 ^b^	−999.13 ± 14.26 ^a^

Results are presented as average ± standard deviation, *n* = 3. The letters represent significant differences between BF and EF samples.

**Table 5:** Hospital bacteria samples from different biological materials used in screening of maceration, infusion, Soxhlet, and ultrasound extracts.

Sample	Bacterium
Tracheal secretion	*Enterobacter cloacae*
Blood	*Enterobacter* sp.
Urine	*Enterobacter agglomerans*
Urine	*Escherichia coli*
Tracheal secretion	*Escherichia coli*
Urine	*Escherichia coli*
Blood	*Klebsiella pneumoniae*
Urine	*Klebsiella pneumoniae*
Urine	*Klebsiella ozaenae*
Catheter tip	*Proteus mirabilis*
Catheter tip	*Acinetobacter*
Urine	*Pseudomonas aeruginosa*

**Table 6:** Components of blank (BF) and *Rosmarinus officinalis* extract (EF) formulations.

Components	Concentration %	
	BF	EF
Sorbitol	5	5
Benzoic acid	0.3	0.3
Xanthan gum	1.6	1.6
Soy lecithin	1.5	1.5
Stearyl alcohol	4	4
Sunflower oil	5	5
Sorbic acid	0.3	0.3
Sodium hydroxide *q.s.p.*	pH 5.5–6.5	pH 5.5–6.5
*Rosmarinus officinalis*	-	5
Water *q.s.p.*	100	100

**Table 7:** Settings and parameters used in textural and spreadability analyses of the blank and extract formulations.

Settings and Parameters	Textural Analyses	Spreadability
Test Mode	Compression	Compression
Pre-Test Speed	1.50 mm/s	1.00 mm/s
Test Speed	2.00 mm/s	3.00 mm/s
Post-Test Speed	2.00 mm/s	10.00 mm/s
T.A. Variable No: 5	0.0 g	-
Target Mode	Distance	Distance
Force	-	100.0 g
Distance	25.000 mm	23.000 mm
Strain	10.0%	10.0%
Trigger Type	Auto (Force)	Button
Trigger Force	30.0 g	5.0 g
Probe	A/BE-d35; back extrusion rig 35 mm disk	HDP/SR; spreadability rig

## Data Availability

The data presented in this study are available upon request from the corresponding author.

## Sample Availability

Samples of the compounds are not available from the authors.
